# Development and Validation of a Risk Score for Predicting Death after Pneumonectomy

**DOI:** 10.1371/journal.pone.0121295

**Published:** 2015-04-09

**Authors:** Seyer Safi, Axel Benner, Janos Walloschek, Maria Renner, Jan op den Winkel, Thomas Muley, Konstantina Storz, Hendrik Dienemann, Hans Hoffmann, Thomas Schneider

**Affiliations:** 1 Department of Thoracic Surgery, Thoraxklinik, Heidelberg University Hospital, Heidelberg, Baden-Württemberg, Germany; 2 Division of Biostatistics, German Cancer Research Center, Heidelberg, Baden-Württemberg, Germany; 3 Department of Thoracic Surgery, St. Vincentius Kliniken Karlsruhe, Karlsruhe, Baden-Württemberg, Germany; Harvard Medical School, UNITED STATES

## Abstract

Pneumonectomy is associated with significant postoperative mortality. This study was undertaken to develop and validate a risk model of mortality following pneumonectomy. We reviewed our prospective database and identified 774 pneumonectomies from a total of 7792 consecutive anatomical lung resections in the years 2003 to 2010 (rate of pneumonectomy: 9.9%). Based on data from 542 pneumonectomies between 2003 and 2007 (i.e., the "discovery set"), a penalized multivariable logistic regression analysis was performed to identify preoperative risk factors. A risk model was developed and validated in an independent data set of 232 pneumonectomies that were performed between 2008 and 2010 (i.e., the "validation set"). Of the 542 patients in the discovery set (DS), 35 patients (6.5%) died after pneumonectomy during the same admission. We developed a risk prediction model for in-hospital mortality following pneumonectomy; that model included age, current alcohol use, coronary artery disease, preoperative leukocyte count and palliative indication as possible risk factors. The risk model was subsequently successfully validated in an independent data set (n = 232) in which 18 patients (7.8%) died following pneumonectomy. For the validation set, the sensitivity of the model was 53.3% (DS: 54.3%), the specificity was 88.0% (DS: 87.4%), the positive predictive value was 26.7% (DS: 22.9%) and the negative predictive value was 95.8% (DS: 96.5%). The Brier score was 0.062 (DS: 0.054). The prediction model is statistically valid and clinically relevant.

## Introduction

Pneumonectomies are performed in selected patients with lung cancer with curative intent if the tumor cannot be removed by lesser resections, such as sleeve lobectomy or segmentectomy. Palliative pneumonectomy might be indicated in cases with e.g., massive hemoptysis or septic conditions secondary to poststenotic pneumonia, in which non-surgical procedures have failed. Both curative and palliative pneumonectomies are associated with significant postoperative mortalities that have been reported to range from 3% to 26% [1–4]. To better select and risk-stratify these patients, several studies have been conducted with the goal of identifying predictors of adverse outcomes.

Laterality of the procedure [5, 6], more extensive surgical resection (e.g., pleuropneumonectomy or pneumonectomy with chest wall resection) [6, 7], urgency of the indication [8] and comorbid diseases (e.g., coronary artery disease, hypertension, underlying lung diseases and active cigarette smoking) have been found to be associated with increased postoperative mortality in many studies. However, there are contradictory findings regarding the predictors of increased postoperative mortality [1–3, 9–11]. For example, some studies have reported that advanced age is a risk factor for increased mortality following pneumonectomy [12–16], while others have not reported this finding [10, 17]. Whether preoperative chemotherapy or radiotherapy increases operative mortality also remains unclear, as studies have found conflicting results [18, 19]. Completion pneumonectomy has also been reported as a predictive risk factor for postoperative death [10]; however, other authors were not able to confirm these findings [20].

Hospital mortality that is associated with inpatient surgery varies widely. Therefore, we sought to first identify the preoperative factors associated with increased in-hospital mortality among patients undergoing pneumonectomy and, subsequently, to develop a risk prediction score that can be applied to predict the risk of in-hospital death for patients who are considered candidates for pneumonectomy.

## Materials and Methods

### Patients and data collection

The prospectively maintained database at our institution was queried for pneumonectomies. From a total of 7792 consecutive anatomical lung resections from January 2003 to December 2010 we identified 774 patients (male: n = 575, female: n = 199) who underwent pneumonectomy (pneumonectomy rate: 9.9% in the same timeframe). The medical records were analyzed retrospectively for possible risk factors for in-hospital mortality, which was defined as death during the same hospitalization. The variables that were analyzed are summarized in Table 1.

**Table 1:** Characteristics of 542 consecutive pneumonectomies from 2003 to 2007 (discovery set).

Variable	n	
Patients total	542	
Median age, years	61	
Male sex	404	(75%)
Laterality of procedure		
Right	245	(45%)
Left	297	(55%)
ASA score		
ASA 1	8	(1%)
ASA 2	426	(79%)
ASA 3	104	(19%)
ASA 4	4	(1%)
FVC (range), liter	3.4	(1.2–6.3)
FEV1 (range), liter	2.4	(0.7–4.9)
FEV1 (range), %	72.3	(30.0–119.1)
Past medical history of malignant disease	56	(10%)
Smoking habits		
Current smoker	70	(13%)
Ex-smoker, cessation less than 6 months ago	157	(29%)
Ex-smoker, cessation more than 6 months ago	152	(28%)
Never smoker	104	(19%)
Missing data	59	(11%)
Arterial hypertension	233	(43%)
Valvular heart disease	18	(3%)
Coronary artery disease	72	(13%)
Peripheral vascular disease	78	(14%)
Diabetes mellitus		
Insulin dependent	16	(3%)
Non-insulin dependent	38	(7%)
Current alcohol use	70	(13%)
Renal insufficiency	19	(4%)
Liver disease	74	(14%)
Liver cirrhosis	4	(1%)
Pulmonary disease	367	(68%)
Preoperative pneumonia	53	(10%)
Median preoperative leukocyte count (range), x 1,000/ μl	8.9	(3.0–36.0)
Preoperative leukocyte count ˃ 13,000/ μl	69	(13%)
Median preoperative hemoglobin (range), g/dl	12.9	(7.9–17.3)
Median preoperative creatinine (range), mg/dl	0.8	(0.1–6.6)
Preoperative chemotherapy	121	(22%)
Preoperative radiotherapy	21	(4%)
Priority of pneumonectomy		
Elective	515	(95%)
Urgent or emergency	27	(5%)
Indication		
Curative	444	(82%)
Palliative	98	(18%)
Type of pneumonectomy		
Standard/intrapericardial	368	(68%)
Carinal resection	30	(6%)
Extrapleural pneumonectomy with diaphragm	58	(11%)
Other extensions*	86	(16%)
Completion pneumonectomy	27	(5%)
Disease category		
Adenocarcinoma of lung	148	(27%)
Squamous cell carcinoma of lung	215	(40%)
Small cell carcinoma of lung	8	(1%)
Metastasis to lung	26	(5%)
Other malignant disease	129	(24%)
Benign disease	16	(3%)
Resection margins		
R0	365	(67%)
R1 or R2	163	(30%)
In-hospital mortality	35	(6%)

ASA = American Association of Anesthesiologists. FEV1 = median forced expiratory volume in 1 second. FVC = median forced vital capacity.

*Pneumonectomies with resections of the atrium, diaphragm, chest wall or superior vena cava were defined as other extensions.

The patients routinely received an epidural catheter preoperatively and underwent endobronchial double-lumen intubation. Anterolateral thoracotomies that involved entering through the fourth or fifth intercostal space were chosen for standard and intrapericardial pneumonectomies. Depending on the tumor location and the expected surgical extension, posterolateral thoracotomies were alternatively performed. Radical mediastinal and hilar lymphadenectomies were performed in all cases of malignancy as previously described [21]. The patients were extubated in the operating room and transferred to the intensive care unit where blood pressure, heart rate, electrocardiographic tracings, respiratory frequency and oxygen saturation were monitored continuously for at least 48 hours.

Data from 542 patients (January 2003 to December 2007) were analyzed to develop a risk prediction model (discovery set). An independent data set of 232 patients (January 2008 to December 2010) was considered the validation set. To balance calendar related changes in the cohort, year of pneumonectomy was selected as variable for analysis.

The complete characteristics of the patients in the discovery set are shown in Table 1. Comorbidities were defined as listed in Table 2.

**Table 2:** Definitions of the comorbidities according to the International Classification of Diseases (ICD) version 10.

Comorbidity	Definition
Current alcohol use	Chronic alcohol consumption up to the time of admission to hospital with an average of more than 1 beverage daily for women and more than 2 alcoholic beverages daily for men over the last 30 days. One beverage is equivalent to 10 grams of ethyl alcohol, which equates to approximately a 330 ml can of beer or a 100 ml glass of wine.
Arterial hypertension	Systolic/ diastolic blood pressure ˃ 140/ 90 mmHg or under medical treatment
Coronary artery disease	Coronary artery disease diagnosed by coronary angiography or previous myocardial infarction treated with percutaneous coronary intervention, surgery or medical treatment
Liver disease	Cirrhosis, decreased pseudo cholinesterase enzyme activity, fatty liver or acute or chronic hepatitis A or B or C

The Ethics Committee of Heidelberg University formally approved this study (study number: 080/2006).

### Statistical analyses

Based on a literature review and clinical judgment, a total of 20 patient characteristics (age, gender, laterality, smoking habits, arterial hypertension, coronary artery disease, peripheral vascular disease, current alcohol use, liver cirrhosis, preoperative pneumonia, preoperative leukocyte count greater than 13,000 / μl, preoperative chemotherapy, preoperative radiotherapy, priority of pneumonectomy, indication, type of pneumonectomy, completion pneumonectomy, disease category, resection margins and year of pneumonectomy) were selected as candidate prediction covariates for the discovery set. To compensate for missing data, multivariable imputation by chained equations [22] utilizing predictive mean matching with 20 multiple imputations was performed to incorporate observations with incomplete variables in the regression models. A “complete case analysis” (n = 458) served as the control for the final model.

To evaluate the candidates for risk prediction we used (i) a logistic regression model with backward variable elimination and (ii) an L1 penalized logistic regression analysis with internal 10-fold cross-validation for model selection [23]. The final risk prediction model was then developed using the final candidates derived from the two approaches. An approximation of the final risk prediction model was then used to define a risk prediction score (RPS).

The performance of the risk prediction model and the ability of the risk prediction score to predict postoperative in-hospital mortality following pneumonectomy were then evaluated in the independent validation set (n = 232). To assess the performance of the final risk prediction model, we considered the discrimination ability and calibration of the model [24]. Calibration refers to the agreement between the observed outcomes and the predictions. The estimated calibration factor indicates the extent to which the predictions are systematically too low or too high. A factor smaller than 1 reflects overfitting of the model. To measure the discrimination ability of the final risk prediction model, the sensitivity, specificity and positive and negative predictive values were computed. Nagelkerke´s R^2^ index was used to describe the goodness of fit of the final model to the discovery and validation sets. Additionally, the c-index was computed to describe the discrimination ability of the model, and the Brier score was used to measure the predictive ability of the model.

All statistical analyses reported in the manuscript were performed in the statistical software environment R, version 2.14.2 (R Development Core Team 2012) using the R packages rms (version 3.5–0) and glmnet (version 1.7.3).

## Results

### Development of the risk prediction model

The observed hospital mortality in the discovery set (n = 542) was 6.5% (n = 35). The causes of death in the discovery set were pneumonia (n = 12), myocardial infarction (n = 7), arrhythmia (n = 4), acute hemothorax (n = 4), postpneumonectomy empyema (n = 3), pulmonary embolism (n = 1), pulmonary edema (n = 1), cardiac herniation (n = 1), bronchopleural fistula (n = 1) and acute renal injury (n = 1). The causes of death in the validation set (n = 18) were pneumonia (n = 5), myocardial infarction (n = 4), postpneumonectomy empyema (n = 3), acute hemothorax (n = 2), bronchopleural fistula (n = 2), arrhythmia (n = 1) and acute liver failure (n = 1). The median postoperative hospital stay was 15 days in the whole cohort and 12 days in the cohort of patients who died. The patients´ baseline, clinical and demographic characteristics and comorbidities are summarized in Tables 1 and 2. Based on our exploratory analysis for the derivation of a risk prediction model, the following five factors were identified as possible risk factors for postoperative in-hospital mortality: age, palliative indication, current alcohol use, preoperative leukocyte count greater than 13,000/ μl and coronary artery disease. The linear predictor of the final logistic regression model resulted as:

$$\begin{array}{l} RPS=-6.152+\left(0.039\times \text{age}\;\left(\text{years}\right)\right)\\ +\left(1.138\times \text{palliative indication}\;\left(\text{no}=0\;\text{or yes}=1\right)\right)\\ +\left(1.318\times \text{current alcohol use}\;\left(\text{no}=0\;\text{or yes}=1\right)\right)\\ +\left(1.276\times \text{preoperative leukocyte count greater than}\;13,000/\mu \text{l}\;\left(\text{no}=0\;\text{or yes}=1\right)\right)\\ +\left(1.039\times \text{coronary artery disease}\;\left(\text{no}=0\;\text{or yes}=1\right)\right) \end{array}$$

(see also Table 3).

**Table 3:** Final risk prediction model of in-hospital mortality after pneumonectomy in the discovery set based on data from 542 consecutive pneumonectomies that were performed between 2003 to 2007.

Variable	Odds ratio	95% CI	*p* value
Age, years	1.04	1.00–1.09	0.07
Indication (palliative vs. curative)	3.12	1.47–6.64	0.003
Current alcohol use (yes vs. no)	3.73	1.63–8.55	0.002
Preoperative leukocyte count higher than 13,000/ μl (yes vs. no) **¶**	3.58	1.61–7.96	0.002
Coronary artery disease (yes vs. no)	2.83	1.18–6.78	0.02

CI = confidence interval.

¶ High leukocyte counts were not considered “yes” if related to steroids or immunosuppression with no clinical signs of infection.

The estimated sensitivity and specificity of this predictor were 54.3% and 87.4%, respectively. The positive predictive value was 22.9%, and the negative predictive value was 96.5% assuming an a priori rate of hospital mortality of 10%. The c-index was c = 0.756, R^2^ was estimated as 0.168, and the Brier score was BS = 0.054.

A risk prediction score (RPS) was calculated by simplifying the final risk prediction model for predicting postoperative death after pneumonectomy as follow:

$$\begin{array}{l} RPS=-6+\left(0.04\times \text{age}\;\left(\text{years}\right)\right)\\ +\left(1.1\times \text{palliative indication}\;\left(\text{no}=0\;\text{or yes}=1\right)\right)\\ +\left(1.3\times \text{current alcohol use}\;\left(\text{no}=0\;\text{or yes}=1\right)\right)\\ +\left(1.3\times \text{preoperative leukocyte count greater than}\;13,000/\mu \text{l}\;\left(\text{no}=0\;\text{or yes}=1\right)\right)\\ +\left(1.0\times \text{coronary artery disease}\;\left(\text{no}=0\;\text{or yes}=1\right)\right) \end{array}$$

### Validation

To predict the probability of postoperative in-hospital death following pneumonectomy, we applied the RPS to the patients in the validation set. The patients´ baseline, clinical and demographic characteristics were retrospectively collected after the development of the risk prediction model. These data are summarized in Table 4.

**Table 4:** Characteristics of 232 consecutive pneumonectomies from 2008 to 2010 (validation set).

Variable	n =	
Patients total	232	
Median age, years	61	
Male sex	171	(74%)
Smoking habits		
Current smoker	58	(25%)
Ex-smoker, cessation less than 6 months ago	40	(17%)
Ex-smoker, cessation more than 6 months ago	97	(42%)
Never smoker	33	(14%)
Coronary heart disease	25	(11%)
Peripheral vascular disease	25	(11%)
Current alcohol use	14	(6%)
Liver cirrhosis	2	(1%)
Preoperative pneumonia	109	(47%)
Preoperative leukocyte count greater than 13,000/ μl	24	(10%)
Preoperative radiotherapy	9	(4%)
Priority of pneumonectomy		
Elective	212	(91%)
Urgent or emergency	20	(9%)
Indication		
Curative	195	(84%)
Palliative	37	(16%)
Resection margins		
R0	161	(69%)
R1 or R2	71	(31%)
In-hospital mortality	18	(8%)

ASA = American Association of Anesthesiologists.

For the predictions, we assumed a 10% a priori rate of hospital mortality in the discovery set. In contrast, in the validation set (n = 232), the hospital mortality was 8% (n = 18). The application of the prognostic score to the validation dataset resulted in a calibration factor of 1.15, which indicated that overfitting did not occur. The c-index and R^2^ for the validation dataset (c = 0.848 and R^2^ = 0.253) were better than those for the discovery set. The Brier score was slightly worse for the validation dataset (BS = 0.062).

For the validation set, the sensitivity of the RPS was 53.3% (discovery set: 54.3%), the specificity was 88.0% (discovery set: 87.4%), the positive predictive value was 26.7% (discovery set: 22.9%) and the negative predictive value was 95.8% (discovery set: 96.5%).

## Discussion

In this study, a risk prediction model for the prediction of postoperative in-hospital death following pneumonectomy was developed based on a cohort of 542 patients (discovery set) and subsequently validated on an independent dataset from an additional 232 patients (validation set).

Different endpoints are used in the literature to calculate the risk of postoperative death [25]. The goal of this study was to develop a tool for communicating the risk of postoperative death to the patient. Therefore the endpoint favoured in this study was in-hospital mortality, defined as death during the same hospitalization, giving the patient an estimate of the chance of going home.

The postoperative in-hospital mortalities of both, the discovery set and the validation set, were within the range that has been reported in the literature [1–4]. We found that age, current alcohol use, preoperative leukocyte count above 13,000/ μl, palliative indication and coronary artery disease were predictors of postoperative death following pneumonectomy (Table 1). Sensitivity, specificity positive predictive value and the negative predictive value in the discovery set were similar to the validation set. These results indicate the reliability and high predictive value of this risk model.

The adverse effect of higher age on postoperative mortality following pneumonectomy corresponds to most but not all of the reports in the literature [10, 12–17]. Coronary artery disease was a predictor of postoperative mortality, which is in agreement with the results of Bernard et al. who analyzed a cohort of 639 pneumonectomies and found that cardiovascular disease was predictive of postoperative mortality [10]. Senbaklavaci et al. reported no differences in postoperative morbidity or mortality following major pulmonary resection for NSCLC between 31 patients with previous cardiovascular disease and 167 patients with no cardiovascular disease history [26]. However, only 5 patients in the subgroup of 31 patients with previous cardiovascular disease underwent pneumonectomies; thus the utility of the comparison of these findings to our own is limited.

Current alcohol use was also found to be predictive of postoperative mortality in our cohort. This finding contrasts those of prior studies of morbidity and mortality following pneumonectomy in which the analyses also included alcohol history, but not strong associations between alcohol intake and postoperative mortality were detected. The fact that the quantities of alcohol consumed were not specified in many of these reports must be taken into account [27]. This discrepancy with our results might be attributable to a lack of standardized documentation for alcohol consumption in clinical studies in general. In our study data regarding the type and amount of alcoholic beverages consumed and the frequency and duration of alcohol consumption were structurally collected. We defined current alcohol use as chronic alcohol consumption up to the time of admission to the hospital that averaged more than 1 beverage daily for women and more than 2 alcoholic beverages daily for men over the previous 30 days (Table 2).

Interestingly, a preoperative leukocyte count greater than 13,000/ μl was strongly associated with an increased risk of postoperative death. A high leukocyte count might be considered a marker of the beginning of septic disease, e.g., due to poststenotic pneumonia, which is consistent with observations of Joo et al. who identified sepsis as a significant mortality factor [28].

We observed that palliative indications were predictive of postoperative death following pneumonectomy. In our review of the literature, we found no data about the associations between palliative and curative indications with death following pneumonectomy. Palliative surgery was indicated for clinical situations, such as significant hemoptysis or septic conditions secondary to poststenotic pneumonia, in which more conservative measures had failed. Given that most of the cases of palliative pneumonectomy were non-electively performed, the predictive value of this parameter accords with the analyses of the Society of Thoracic Surgeons General Thoracic Database of the predictors of mortality and major morbidity for lung cancer resection that identified the urgency of the procedure as a predictor of mortality [8, 16]. This raises the question whether patients known to have conditions that are likely to require pneumonectomy could be considered for surgery earlier in the disease course, before developing complicating acute problems. However, the timing of pneumonectomy in urgent or palliative cases was not the focus of this study.

Contrary to our expectations, some factors previously identified to be predictive of complicated recovery, such as smoking habits, laterality of the pneumonectomy, chronic obstructive lung disease, extended resection and neoadjuvant radiotherapy [1–3, 7, 9–11, 14, 15, 28–30] were not identified as risk factors by this analysis. The knowledge of previously identified risk factors may have resulted in special surgical techniques and perioperative care. For example, we routinely cover the bronchial stump with a pericardial flap after pneumonectomy on the right side, which may have resulted in no influence of laterality on bronchial stump insufficiency rates. Added attention to preoperative strategies, such as optimizing COPD or offering smoking cessation support, might have improved outcomes. These measures have been conducted in our hospital for many years. A strong statistical association does not necessarily indicate a cause and effect relationship and risk factors may be modifiable why previously reported risk factors might not have been confirmed in this study. Table 5 provides an overview of the predictive risk factors for mortality following pneumonectomy that have been reported in the literature.

**Table 5:** Selected studies of preoperative risk factors for mortality after pneumonectomy.

Author	Year	Patients	Mortality (%)	Risk factors	Endpoint
Patel et al.	1992	197	8.6	Coexisting medical conditions, FEV1/ FVC less than 0.55	In-hospital mortality
Bernard et al.	2001	639	7.0	Associated cardiovascular or hematologic disease, hemoglobin level, neoadjuvant chemotherapy, DLCO, laterality, extended resection, completion pneumonectomy, crystalloid infusion, bronchial stump reinforcement	Postoperative mortality
Alexiou et al.	2001	206	6.8	Age	In-hospital mortality
Mansour et al.	2009	323	3.4–8.3	Laterality, chronic obstructive lung disease	30- and 90-day mortality
Fernandez et al.	2011	9746	4–16	Laterality	30- and 90-day mortality
Bagan et al.	2013	86	5.8	Malnutrition, extended resection	90-day mortality

FEV1/ FVC = forced expiratory volume in 1 second/ forced vital capacity; DLCO = diffusion capacity of lung to carbon monoxide.

Large cohort database analyses have yielded contradictory results [16, 31–33]. These findings are based on data from major lung resections in general, and the endpoints were morbidity and mortality. However, the results regarding the perioperative risk factors based on these database analyses should be interpreted with caution because it might be inappropriate to extrapolate these results to patients undergoing pneumonectomy, which is associated with high risks of postoperative morbidity and mortality.

Another limitation of this study is the retrospective design, even though the data were collected prospectively, and the risk prediction score (RPS) was developed independently of the second cohort. The study spanned nearly 7 years. Regardless of changes in intensive care management, chemotherapy and radiotherapy regimes, this RPS was validated in an independent validation set.

There was no risk assessment model in thoracic surgery until the Thoracoscore risk model for in-hospital mortality was published in 2007 [33]. The Thoracoscore model is based on data from 15183 patients and has reached international acceptance. However, in a recent study that included 243 pneumonectomies the Thoracoscore model failed to predict accurate risk of in-hospital mortality [34]. An explanation for the inability of the Thoracoscore model to predict mortality accurately in this high-risk subgroup might be that all thoracic surgical procedures were included and only 6% in the Thoracoscore cohort underwent pneumonectomy.

Our model addresses only in-hospital mortality, which is an imperfect surrogate for the risk of death attributable to surgery. Thirty-day mortality may underestimate the risk of death attributable to the operation [35], whereas in-hospital mortality is susceptible to a so-called "discharge bias" if early post-discharge mortality is not included in the calculation of mortality. It is important to note, that patients, who developed postoperative complications, did not require transfer from our hospital to another facility. Therefore, all patients, who died due to postoperative complications, were included in the calculation of in-hospital mortality.

Although our risk prediction model may identify patients at risk, it cannot predict in which single individual a complication may develop and therefore should not be used to exclude patients from surgery.

The practice at our institution has not changed during the timeframe of this study. However, it is possible that practices have evolved in other centers, which makes the utility of this index reliable in a population, but of limited applicability to any individual patient. The predictive value of the risk prediction score presented in this study may be limited by the fact that our model is insufficiently transportable to warrant its use for patients at other centers, hence generalizations need to be undertaken with caution

In conclusion, in this large cohort of patients who underwent pneumonectomies, we identified predictors of the risk for death following surgery. This data might be useful in improving the information that is given to patients and enable forward planning of the management of those, who are considered to be most at risk.
